# Evaluating the safety and efficacy of equine peripheral blood-derived mesenchymal stem cells in feline chronic gingivostomatitis

**DOI:** 10.3389/fvets.2026.1857014

**Published:** 2026-06-15

**Authors:** Nathalie de Bouvre, Charlotte Beerts, Sarah Y. Broeckx, Eva Depuydt, Liesa Tack, Glenn Pauwelyn, Luc Duchateau, Jimmy Saunders, Jan H. Spaas

**Affiliations:** 1 DAP de Molenkreek, Westdorpe, Netherlands; 2 UniLaSalle, Université d’Artois, Mont-Saint-Aignan, France; 3 Research and Development Department, INTIBIO, Bree, Belgium; 4 Boehringer Ingelheim Veterinary Medicine Belgium, Evergem, Belgium; 5 Biometrics Research Center, Faculty of Veterinary Medicine, Ghent University, Merelbeke, Belgium; 6 Department of Morphology, Imaging, Orthopedics, Rehabilitation and Nutrition, Faculty of Veterinary Medicine, Ghent University, Merelbeke, Belgium

**Keywords:** chronic, ePB-MSC, feline, gingivostomatitis, xenogeneic

## Abstract

**Introduction:**

Feline chronic gingivostomatitis (FCGS) is a painful chronic disease with limited treatment options, especially in severe cases. For this reason, regenerative therapies are being explored as a potential solution.

**Methods:**

The current study investigates the safety and efficacy of systemic application of equine peripheral blood-derived mesenchymal stem cells (ePB-MSCs) in 16 cats with feline chronic gingivostomatitis (FCGS). A blinded, randomized, placebo-controlled study was performed in one veterinary practice with daily owner evaluations and veterinary visits on days −7, 0, 21 ± 3, 42 ± 3, and 84 ± 3 (optional: 182 ± 5).

**Results:**

In both treatment groups, one adverse event and one serious adverse event were reported, which were not linked to the treatment. Hematology and biochemistry assessments did not result in any remarkable changes. The change in Stomatitis Disease Activity Index Score (SDAI) did not show a significant effect for time (*p* = 0.368), treatment (*p* = 0.626), or the interaction (*p* = 0.696). There is no significant difference with respect to the failure time in the two treatment groups (*p* = 0.629) or the number of animals receiving rescue therapy in the two treatment groups (*p* = 0.26). Moreover, biopsy analysis did not result in a significant difference over time (*p* = 0.386). Furthermore, the average owner reports did not show any effect of time (*p* = 0.631).

**Discussion:**

In conclusion, the single intravenous application of ePB-MSCs in cats suffering from FCGS was safe. Based on the current data using the current study design, there was no evidence that the application was more effective than the control. Further studies evaluating the efficacy of equine peripheral blood-derived mesenchymal stem cells in cats suffering from chronic gingivostomatitis with a longer follow-up, different evaluation parameters, and/or adjusted treatment regimen are recommended.

## Introduction

Feline chronic gingivostomatitis (FCGS) is a painful and debilitating disease with serious implications for the quality of life of the affected animal (1–3). The disease is typically associated with severe inflammatory lesions of the oral cavity, which can be erosive and/or proliferative in nature. In contrast to traditional gingivitis and periodontal disease, the lesions are not only located on the gingiva and the alveolar region, but also on the labio-buccal and caudal oral mucosa, with a typical distribution lateral to the palatoglossal folds (1, 3, 4). FCGS has a protracted character and can last for months or even years. Associated clinical symptoms include weight loss, oral pain, loss of appetite, halitosis, ptyalism, and reduced grooming (1, 3). Severely affected cats are often euthanized (5).

The etiology of FCGS is not fully understood; nevertheless, an inappropriate response of the patient’s immune system to chronic oral antigenic stimulation of both infectious and non-infectious origin is postulated (1–3). Examples of possible causative agents are viruses (e.g., feline calicivirus, feline leukemia virus, and feline immunodeficiency), bacteria (e.g., *Bartonella* sp. and *Pasteurella multocida*), dental diseases, food allergies, and an oversensitivity reaction to dental plaque bacteria (1, 3). Despite this plethora of possible causal factors, the histologic image of affected areas always includes a lymphoplasmacytic infiltration of the mucosa and the submucosa, indicating a chronic involvement of viral factors (1, 5–7).

Because FCGS is multifactorial, its treatment proves to be challenging. Current pharmacological treatment consists of immunosuppressive (e.g., corticosteroids) or anti-inflammatory drugs [e.g., non-steroidal anti-inflammatory drugs (NSAIDs)], often combined with antimicrobials (e.g., interferon and antibiotics) (3, 4). The standard of care consists of partial or full mouth tooth extraction, with success rates of 70–80% (1, 3, 5, 6). This still leaves 20–30% of the cats refractory to treatment.

Recently, the use of autologous and allogeneic mesenchymal stem cells (MSCs) has been investigated as a treatment for FCGS in cats, which are refractory to tooth extraction. MSCs have the ability to differentiate into different cell lineages, could have an immunomodulatory effect, and could stimulate local repair cells by paracrine signals (8–10). The potential of reducing circulating T-cell, B-cell, natural killer cell, and dendritic cell levels creates an interesting treatment option, especially for refractory immune-mediated inflammatory diseases such as FCGS (11). Promising results are seen with a clinical remission or substantial improvement in over 70% of the treated cats suffering from chronic, non-responsive FCGS using autologous or allogeneic adipose tissue-derived MSCs (5, 6, 12).

Autologous cell treatment proves to be difficult, since 55% of cell lines (with a range of 20–80%) derived from client-owned cats develop syncytial cells and go into proliferation arrest (13). Arzi et al. (13) reported this phenomenon, which may be due to an infection of the cell lines with feline foamy virus (FFV) and could be resolved by using lower cell passages (up to passage 3), since these do not show any adverse effect of the FFV infection. Arzi et al. (13) also commented on the safety issues of using cell lines with active viral infections in patients. Allogeneic cells isolated from specific pathogen-free cats or xenogeneic cells are a more appealing option. These treatment options preclude the need for harvesting tissue from an already compromised patient and enable the development of an “off-the-shelf” treatment, which can be used immediately.

Moreover, as in other veterinary species, feline MSC quality declines with age, so allogeneic or xenogeneic MSCs would allow a stringent donor selection to ensure high-quality stem cells (14). Commercial application of allogeneic feline MSCs is impeded because even FFV-free cell lines show a limited proliferation capacity compared to equine and human MSCs, and the need for specific pathogen-free cats increases the production costs (13, 15). Moreover, blood harvesting from large species such as horses is a straightforward process, and only limited diseases have been reported to transmit between equine blood to cats. For all these reasons, equine peripheral blood-derived MSCs (ePB-MSCs) present an interesting alternative to treat FCGS. Currently, studies investigating the safety and efficacy of ePB-MSCs in cats are lacking.

In the current study, the safety and efficacy of ePB-MSCs as a treatment for cats affected with FCGS and refractory after full mouth tooth extraction and immunosuppressive therapies will be investigated.

## Materials and methods

### Study design

The current study was a randomized, placebo-controlled, and blinded clinical trial evaluating the efficacy and safety of the investigational veterinary product (IVP) equine peripheral blood-derived mesenchymal stem cells (ePB-MSCs) in the treatment of FCGS in cats under field conditions. Cells were isolated from the peripheral blood of a donor horse using Percoll gradient centrifugation and cultured up to passage 10 as previously described (16, 17). The active substance consisted of 3 ×10^5^ cells/mL equine peripheral blood-derived mesenchymal stem cells (1 mL) before freezing, resuspended in 0.9 mL Dulbecco’s Modified Eagle Medium (DMEM) low glucose/mL, and cryopreserved with 10% dimethylsulfoxide (DMSO) and characterized as previously described (16). After thawing the cryovial in the palm of a hand, the suspension was visually assessed for transparency and clearness and immediately injected using a 22 G i.v. catheter (see Table 1).

**Table 1:** Study design.

Stratum	Treatment		Name of product	Application route	Dosage	Day of treatment	Days of observation	Number of animals
A	T^1^	IVP group	ePB-MSCs	IV	1.0 mL	Day 0	−7, 0, 21 ± 3, 42 ± 3, 84 ± 3, (optional: 182 ± 5)	6
	T^2^	CP group	Vetivex 9 mg/ml (NaCl)					3
B	T^1^	IVP group	ePB-MSCs					5
	T^2^	CP group	Vetivex 9 mg/ml (NaCl)					2

The study was controlled by a placebo control product (CP) group receiving Vetivex 9 mg/mL (Dechra Limited, Staffordshire ST7 1XW, UK), applied at the same volume (1.0 mL) and administration route (IV) as the IVP group. The use of a placebo control group was necessary to identify the percentage of animals with insufficient or no response to treatment and spontaneous recovery.

The cats suffering from FCGS with a periodontitis score of maximum 4 (according to the Periodontal Disease Classification of the American Veterinary Dental College) and no external inflammatory or replacement root resorption were included in stratum A, and the cats suffering from FCGS non-responsive to premolar and molar tooth extraction or full-mouth tooth extraction performed at least 6 months prior to enrollment were included in stratum B.

This study was conducted according to European and national regulatory requirements and in compliance with the following documents:

- Belgian animal welfare and VMP legislation as applicable to this product and study
- Ethics committee (LA1700607) guidelines as approved in EC_2020_001 and in the deontological derogation DWZ-KF-20-1.15.56
- Directive 2010/63/EU of the European Parliament and of the Council of 22 September 2010 on the protection of animals used for scientific purposes
- Directive 2001/82/EC as amended
- VICH GL9 (Good Clinical Practice, June 2000)
- EMA/CVMP/EWP/81976/2010: Guideline on Statistical Principles for Veterinary Clinical Trials, as applicable.

### Inclusion and exclusion criteria

Animals were selected on (or before) day 7. Only animals that complied with all of the inclusion criteria and for which none of the exclusion criteria applied were selected for enrolment in the study.

The following inclusion criteria were applied to each animal:

- Signed owner informed consent, i.e., owner willing to sign informed consent and able to attend scheduled appointments and closely follow-up cat during the entire study period.
- Feline chronic gingivostomatitis (FCGS) with a periodontitis score of maximum (≤) 4 (according to the Periodontal Disease Classification of the American Veterinary Dental College) and no external inflammatory or replacement root resorption (stratum A) or FCGS non-responsive to premolar and molar tooth extraction or full-mouth tooth extraction performed at least 6 months prior to enrollment (stratum B) as confirmed by a Stomatitis Disease Activity Index of minimal 5 (i.e., SDAI ≥ 5).
- Absence of dental root remnants or other underlying lesions causative for mouth pathologies on X-ray (can be an old radiograph taken after full-mouth teeth extraction).
- Age ≥12 months.
- Cat was in good general health status apart from the target disease, based on general physical examination on day 0 and medical history for the last 2 months.
- Cats tested negative for feline immunodeficiency virus (FIV) and feline leukemia virus infection (FeLV).
- At least 6 weeks after any surgery in general.
- At least 3 weeks after the last vaccination.
- After a washout period of administered permissible medication before enrollment (see Table 2).

**Table 2:** Permissible and non-permissible concomitant treatments.

Treatments	Product class/name	Time limits for restriction
Restricted (non-allowed) products and procedures before study start and during study	Mesenchymal stem cells (MSCs)	Animals that were ever treated with MSCs should not be enrolled in the study.
	SAIDs	If short acting, either route (e.g., prednisolone): 14 days.
		If topical: 14 days.
		If long acting, either route (e.g., dexamethasone): 28 days.
		If depot application: 56 days.
	Vaccinations	Within 3 weeks prior to day 0.
		During the study period.
	NSAID’s	Within 7 days prior to day 0.
		During the study period.
Allowed products and procedures before the study started and during the study	Sedatives	Allowed during study period for sedation (e.g., for radiographic assessment (RX), sampling).
	Opioid derivatives	Allowed during the study period as rescue medication (e.g., methadone, buprenorphine, or equivalent).

Animals that met one of the following exclusion criteria were not enrolled in the study:

- Age <12 months.
- Cats that were pregnant, lactating, or intended for breeding during the study period.
- Presence of dental roots (remainders) on X-ray.
- Stomatitis Disease Activity Index (SDAI) score of <5.
- Any severe medical condition that, in the opinion of the investigator, would compromise the patient’s safe participation in the study.
- Any condition, actual or anticipated, that the Investigator feels would restrict or limit the patient’s successful participation for the duration of the study.
- Previous participation in a stem cell study.
- Animals that received vaccinations within 3 weeks before day 0.
- Animals that received non-permissible treatments (see Table 2).

### Blinding and randomization method

The study was blinded in the sense that personnel involved in the evaluation were unaware of the treatment received. Therefore, two separate teams were set up, one team was responsible for the clinical examinations, blood work, and biopsy readings (examining vet, laboratory personnel, and pathologist), and the other team for the administration of treatments (dispenser).

The statistician was responsible for preparing and providing the randomization procedure documentation. Two randomization lists were provided to the non-blinded study personnel, one for stratum A and one for stratum B. Within each stratum, blocks of size 3 were used, with in each block two animals assigned to IVP and one animal to CP.

### Study protocol

All owners had to sign an informed consent before the study started, and all animals needed to comply with the aforementioned inclusion and exclusion criteria.

The owner documented a daily observation of the animal throughout the study period, with the optional completion of an owner diary to make sure adverse event reports or disease progression were properly documented. Veterinary controls are documented in Table 3.

**Table 3:** Schedule of events.

Day procedure	Time point of evaluation (days)							
	Day −7 (or earlier)	Day 0 prior to treatment	Day 0 treatment	Day 0 after treatment	Day 21 ± 3	Day 42 ± 3	Day 84 ± 3	Day 182 ± 5
General physical examination		X		X	X	X	X	(X)
Radiographic assessment		X						
Stomatitis Disease Activity Index (SDAI)		X			X	X	X	(X)
Injection site observation		X			X	X	X	(X)
Blood sampling		X			X	X	X	(X)
Calicivirus/feline coronavirus swab		X						
Biopsy sampling		X					X	
Owner report	X	X	X		X	X	X	(X)
Treatment IVP/CP			X					

X: mandatory control; (X): optional control.

### Safety evaluation

Safety was evaluated by general clinical evaluation, injection site evaluation, together with assessing the incidence of (serious) adverse events, suspected adverse drug reactions, and/or abnormal general clinical signs.

Complete blood count (CBC) and serum biochemistry (including SAA, urea, and alpha, beta, and gamma globulins) were performed.

### Efficacy evaluation

Efficacy was evaluated by means of the change in Stomatitis Disease Activity Index (SDAI, Table 4) scores, according to Arzi et al. (12), failure time, days with rescue therapy, biopsy score [0 = no, 1 = mild, 2 = moderate and 3 = severe inflammation, Harley et al. (18)], and the owner report (Table 5). Cats that were prematurely withdrawn from the study due to lack of efficacy and/or the need for a non-allowed rescue therapy were considered as failures (i.e., withdrawal due to target disease).

**Table 4:** Stomatitis Disease Activity Index (SDAI) score.

Scored parameter	0	1	2	3
Average of the owner evaluation	Average of the scored values of the owner report			
Body weight (compared to most recent visit)	Gain ≥0.5 kg	Gain ≥0.25 kg but <0.5 kg	<0.25 kg gain but ≤0.5 kg loss	Weight loss ≥0.5 kg
Maxillary buccal mucosal inflammation	None	Mild	Moderate	Severe
Mandibular buccal mucosal inflammation	None	Mild	Moderate	Severe
Maxillary attached gingival inflammation	None	Mild	Moderate	Severe
Mandibular attached gingival inflammation	None	Mild	Moderate	Severe
Molar salivary gland inflammation	None	Mild	Moderate	Severe
Inflammation of areas lateral to the palatoglossal folds	None	Mild	Moderate	Severe
Oropharyngeal inflammation	None	Mild	Moderate	Severe
Lingual and/or sublingual inflammation	None	Mild	Moderate	Severe
Total score (/30)	Total SDAI score			

**Table 5:** Owner report score.

Score	Degree of appetite
0	Eating normally
1	Eating wet and dry food, but less than a normal amount
2	Eats wet food on his/her own; cannot eat dry food
3	Eats only pureed food, or only when hand fed

Score	Degree of grooming behavior
0	Grooming normally
1	Grooming excessively
2	Grooms occasionally, but not at the ‘pre-illness’ level
3	Will not groom

Score	Degree of activity
0	Normal activity level (playful and active)
1	Plays spontaneously, but not frequently
2	Low activity level, but will play occasionally when engaged by people or other pets
3	No interest in people or other pets, spends most of the time sleeping

Score	Present comfort level
0	Most comfortable (no pain)
1	Mild pain
2	Moderate pain
3	Most painful

### Statistical analysis

For safety assessment, the mean score per animal was first determined and then compared between IVP and CP using the nonparametric Wilcoxon rank sum test. The relatively small sample size in each group limits the statistical power of the comparative tests and the robustness of the conclusions. Nevertheless, analyses can be carried out using non-parametric tests suitable for small sample sizes. The results should, however, be interpreted with caution and regarded as exploratory.

For efficacy assessment, the change of the SDAI score from baseline was compared between the two treatment groups by a mixed model with animal as a random effect and time, treatment, and their interaction as categorical fixed effects factors. The normal distribution assumption was tested by the Shapiro–Wilks test. Failure time was compared between the two treatment groups by the log-rank test. The percentage of days without rescue therapy was compared between IVP and CP by the Wilcoxon rank sum test. The change in the Harley score from day 0 to day 84 was compared between the two treatment groups by the Wilcoxon rank sum test. The analysis was performed without and with last observations carried forward (LOCF). The change of the average owner report score from baseline was analyzed using a nonparametric longitudinal analysis and also applying last observation carried forward (LOCF) to account for missing values.

## Results

### Number of animals included

In total, 16 animals were enrolled in the study and were all used for safety and efficacy evaluation. The animals were privately owned cats, and animal details are reflected in Table 6. As there were no major protocol deviations, no data were excluded.

**Table 6:** Animal details.

Animal ID	Breed	Age	Sex	Neutered
FS01	European Shorthair	1 year	Female	Yes
FS02	European Shorthair	1 year	Female	Yes
FS03	Maine Coon	2 years	Female	No
FS04	European Shorthair	9 years	Female	Yes
FS05	European Shorthair	5 years	Female	Yes
FS06	European Shorthair	7 years	Female	Yes
FS07	Maine Coon	2 years	Female	No
FS08	American Curl	7 years	Male	Yes
FS09	European Shorthair	12 years	Male	Yes
FS10	British shorthair	2 years	Female	Yes
FS11	Maine Coon	11 years	Female	Yes
FS12	European shorthair	8 years	Female	Yes
FS13	European shorthair	11 years	Female	Yes
FS14	British shorthair	4 years	Male	No
FS15	Maine Coon	3 years	Male	No
FS16	Maine Coon	1 year	Female	No

A total of 11 animals were randomly assigned to the IVP, and five to the CP. Specific baseline variables were compared between the two groups, and no significant differences were found.

### Safety evaluation

#### Incidence of abnormal general clinical signs

No abnormalities were observed in the two treatment groups throughout the whole study period for capillary filling time, body condition score, respiration rate, body weight, behavior, attitude, cardiovascular system, mucous membranes, and gastrointestinal system.

Different clinical response variables can be found in Table 7 and compared between treatment groups. The table includes the animal numbers deviating from the normal score of 0.00. The mean presence was shown in order to cope with the missing values. None of the remaining general clinical signs, i.e., Attitude, Behavior, Lymphatic system, Pulse Rate, Respiratory Rate, Respiratory Tract, Skin/Hair, and Temperature, showed a significant difference between the two treatment groups.

**Table 7:** Overview of number of animals with a particular mean score for the general clinical signs variables for which events were observed in the two treatment groups.

Clinical signs	C								T								*p*-value
respvar	0.00	0.20	0.33	0.40	0.60	0.67	0.80	1.00	0.00	0.20	0.33	0.40	0.60	0.67	0.80	1.00	
Attitude	1	4	.	.	.	.	.	.	0	11	.	.	.	.	.	.	0.249
Behavior	1	4	.	.	.	.	.	.	0	11	.	.	.	.	.	.	0.249
Lymphatic system	5	.	.	.	.	.	.	0	10	.	.	.	.	.	.	1	1
Pulse rate	1	1	0	2	1	.	.	.	1	2	1	4	3	.	.	.	0.664
Respiratory rate	2	1	1	1	.	0	.	.	3	4	0	3	.	1	.	.	0.892
Respiratory tract	5	0	.	.	0	.	.	.	9	1	.	.	1	.	.	.	0.895
Skin/hair	5	0	.	.	.	.	0	.	9	1	.	.	.	.	1	.	0.895
Temperature	3	2	0	0	.	.	.	.	5	3	1	2	.	.	.	.	0.682

#### Incidence of adverse events

An adverse event was observed in one animal in the CP group and one animal in the IVP group. The animal in the CP group was diagnosed with giardiasis with mild severity. The animal treated with metronidazole fully recovered after 10 days. The animal in the IVP group was diagnosed with bronchiolitis with mild severity. The animal was treated with amoxicillin/clavulanic acid and fully recovered after 4 days. It was unlikely that the condition was related to the study medication.

A serious adverse event was observed in one animal in the CP group and one animal in the IVP group. The animal in the CP group died after 37 days of introduction into the study. An autopsy and necropsy were performed, and it was concluded that hypertrophic cardiomyopathy was the cause of the death of the animal. The animal in the IVP group died after 29 days of introduction into the study. An autopsy and necropsy were performed, and it was concluded that the poor health condition and several chronic conditions the cat was suffering from could have caused the death of the animal.

No suspected adverse drug reactions were observed since the two adverse events and the two serious adverse events were scored as not being related to the drug. No events were observed in the two treatment groups throughout the whole study period concerning skin abnormalities or heat, pain, pressure, or swelling at the injection site.

#### Hematology and biochemistry

Concerning hematological and serum biochemistry parameters, for each animal, the mean presence of a value out of the limits was derived. Table 8 summarizes the number of animals having a particular mean presence. The table includes the animal numbers deviating from the normal score of 0.00.

**Table 8:** Overview of the number of animals with a particular mean presence of values out of the limits for the blood and serum chemistry variables according to treatment group.

Blood variables	Control							Trt							*p*-value
respvar	0.00	0.25	0.33	0.50	0.67	0.75	1.00	0.00	0.25	0.33	0.50	0.67	0.75	1.00	
A/G	4	.	.	.	.	0	1	6	.	.	.	.	1	4	0.594
Albumin (%)	3	.	.	.	.	1	1	3	.	.	.	.	2	6	0.257
Albumin (g/L)	3	0	.	0	.	1	1	0	2	.	1	.	3	5	0.129
Alkaline phosphatase (U/L)	4	1	.	0	.	.	.	9	1	.	1	.	.	.	0.771
Alpha 1 globulins (%)	1	2	.	1	.	1	0	1	1	.	1	.	5	3	0.152
Alpha 1 globulins (g/L)	1	1	.	2	.	1	0	1	1	.	3	.	4	2	0.330
Alpha 2 globulins (%)	1	1	.	2	.	.	1	6	2	.	2	.	.	1	0.249
Alpha 2 globulins (g/L)	1	1	.	2	.	.	1	6	2	.	2	.	.	1	0.249
Aspartate transaminase (U/L)	5	0	.	.	.	0	.	9	1	.	.	.	1	.	0.895
Atypical cells (%)	5	.	.	.	.	.	.	11	.	.	.	.	.	.	1
Band neutrophils (absolute)	.	.	.	.	.	.	5	.	.	.	.	.	.	11	1
Basophiles (absolute)	5	0	.	.	.	.	.	10	1	.	.	.	.	.	1
Beta 1 globulins (%)	2	2	.	6	.	.	.	12	10	.	0	.	.	.	0.147
Beta 1 globulins (g/L)	1	0	.	3	.	1	.	4	4	.	3	.	0	.	0.181
Beta 2 globulins (%)	5	0	.	0	.	.	.	9	1	.	1	.	.	.	0.895
Beta 2 globulins (g/L)	5	.	.	.	.	.	.	11	.	.	.	.	.	.	1
Bile acids (μmol/L)	5	.	.	.	.	.	.	11	.	.	.	.	.	.	1
Bilirubin (μmol/L)	5	.	.	.	.	.	.	11	.	.	.	.	.	.	1
Creatinine (μmol/L)	4	.	.	1	.	.	.	11	.	.	0	.	.	.	0.249
Eosinophils (absolute)	3	0	1	1	.	.	.	8	2	0	1	.	.	.	0.377
Erythrocytes (T/L)	1	.	1	1	0	1	1	1	.	3	1	1	2	3	0.737
Fibrinogen (g/L)	3	1	.	.	1	0	0	6	2	.	.	0	1	2	0.955
Gamma globulins (g/L)	5	.	.	.	.	.	.	11	.	.	.	.	.	.	1
Gamma glutamyl transferase (U/L)	5	.	.	.	.	.	.	11	.	.	.	.	.	.	1
Glucose (mmol/L)	3	2	.	0	.	.	.	5	4	.	2	.	.	.	0.684
Hemoglobin concentration (g/dL)	0	0	2	1	0	1	1	1	1	2	0	2	2	3	0.813
Leukocytes (g/L)	3	1	.	0	1	.	.	9	0	.	2	0	.	.	0.331
Lymphocytes (/μL)	2	1	.	1	1	.	.	7	1	.	3	0	.	.	0.341
MCH (pg)	5	.	.	.	.	.	.	11	.	.	.	.	.	.	1
MCHC (g/dL)	1	1	0	1	1	1	0	3	5	1	0	1	0	1	0.354
MCV (fL)	1	1	0	1	1	1	0	3	5	1	0	1	0	1	0.354
Monocytes (/μL)	1	2	1	1	.	0	0	5	3	0	0	.	2	1	0.647
Packed cell volume (%)	5	0	0	0	.	.	.	6	2	1	2	.	.	.	0.395
Polychromasia	0	2	1	2	.	.	.	7	2	0	2	.	.	.	0.081
Segmented neutrophils (/μL)	3	0	1	0	1	.	.	7	1	0	2	1	.	.	0.655
Serum amyloid A (mg/L)	3	1	.	0	.	1	0	6	0	.	1	.	0	4	0.698
Thrombocytes (G/L)	3	1	1	.	.	.	.	9	2	0	.	.	.	.	0.280
Total protein (g/L)	5	.	.	.	.	0	.	10	.	.	.	.	1	.	1
Urea (BUN) (mmol/L)	3	1	.	1	.	.	.	9	2	.	0	.	.	.	0.280

### Efficacy evaluation

#### Stomatitis Disease Activity Index change

An overview of the SDAI scores at the different time points was given in Table 9. The SDAI response variable was first tested for the normal distribution assumption, which was valid according to the Shapiro–Wilk test for both SDAI (*p* = 0.677) and the SDAI change from baseline (*p* = 0.830). For SDAI change from baseline, the overall analysis showed no significant effects for treatment (*p* = 0.626), time (*p* = 0.368), or the interaction between the two factors (*p* = 0.696).

**Table 9:** Overview of the SDAI scores throughout the study.

Animal ID	Stratum	Treatment	D0	D21	D42	D84
FS01	A	IVP	15	18	15	13
FS02	A	IVP	12	9	9	9
FS03	A	CP	10	16	10	13
FS04	A	IVP	13	11	13	14
FS05	B	CP	7	5	5	5
FS06	B	IVP	22	18	/	/
FS07	B	IVP	11	13	10	13
FS08	B	IVP	13	13	12	20
FS09	B	IVP	13	9	11	4
FS10	A	CP	5	4	3	2
FS11	A	IVP	13	15	8	12
FS12	B	CP	13	11	8	13
FS13	B	IVP	11	14	17	18
FS14	A	IVP	5	5	5	5
FS15	A	CP	5	3	/	/
FS16	A	IVP	7	11	10	6

**Figure 1:**
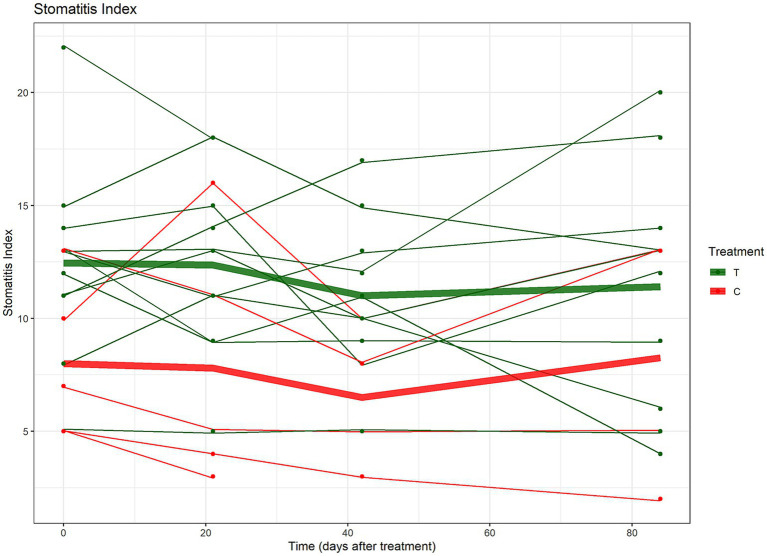
Stomatitis Disease Activity Index Score (SDAI) as a function of time and treatment. Thin lines represent individual scores, whereas thick lines are the means per treatment group.

The comparison between treatment and control overall and at each of the time points is given in Figure 1; Table 10.

**Table 10:** Stomatitis Disease Activity Index Score (SDAI) as a function of time and treatment and change from baseline without and with last observation carried forward (LOCF).

	Time after treatment									
	0		21 ± 3		42 ± 3		84 ± 3		Mean	
	CP	IVP	CP	IVP	CP	IVP	CP	IVP	CP	IVP
SDAI										
Mean	8.0	12.5	7.8	12.4	5.7	11.6	7.5	12.0	7.2	12.1
Stand. error	2.0	1.4	2.0	1.4	2.1	1.4	2.1	1.4	1.8	1.2
95% CI Lo	3.8	9.6	3.6	9.5	1.3	8.8	3.1	9.2	3.4	9.5
95% CI Up	12.2	15.3	12.0	15.2	10.1	14.5	11.8	14.9	11.1	14.7
SDAI LOCF										
Mean	8.0	12.5	7.8	12.4	5.8	11.6	7.2	12.0	7.2	12.1
Stand. error	2.0	1.3	2.0	1.3	2.0	1.3	2.0	1.3	1.8	1.2
95% CI Lo	3.9	9.7	3.7	9.6	1.7	8.9	3.1	9.2	3.4	9.6
95% CI Up	12.1	15.2	11.9	15.2	9.9	14.4	11.3	14.8	11.0	14.7

	CP	IVP	CP	IVP	CP	IVP	CP	IVP
SDAI Δ Baseline								
Mean	−0.2	−0.1	−2.5	−0.7	−0.7	−0.3	−1.1	−0.4
Stand. error	1.5	1.0	1.7	1.1	1.7	1.1	1.3	0.9
95% CI Lo	−3.4	−2.2	−5.9	−2.9	−4.2	−2.5	−3.9	−2.2
95% CI Up	3.0	2.0	1.0	1.5	2.7	1.9	1.6	1.5
*p*-value	0.954		0.381		0.932		0.626	
SDAI Δ Baseline LOCF								
Mean	−0.2	−0.1	−2.2	−0.8	−0.8	−0.5	−1.1	−0.5
Stand. error	1.5	1.0	1.5	1.0	1.5	1.0	1.3	0.8
95% CI Lo	−3.3	−2.2	−5.3	−2.9	−3.9	−2.5	−3.7	−2.3
95% CI Up	2.9	2.0	0.9	1.3	2.3	1.6	1.6	1.4
*p*-value	0.953		0.455		0.851		0.690	

Even at baseline, the control group had a substantially lower SDAI average. Overall, SDAI was significantly lower in the control group compared to the treated group, with no significant differences found at the different timepoints (comparison-wise significance level equal to 0.05/4 = 0.0125). Overall, the mean curves were not changing substantially over time in the two groups, and the differences over time are typically much smaller than the observed difference between the two treatment groups at baseline, i.e., before the treatment was applied. The analysis with last observation carried forward (LOCF) presented basically the same information and is reflected in Table 10.

**Figure 2:**
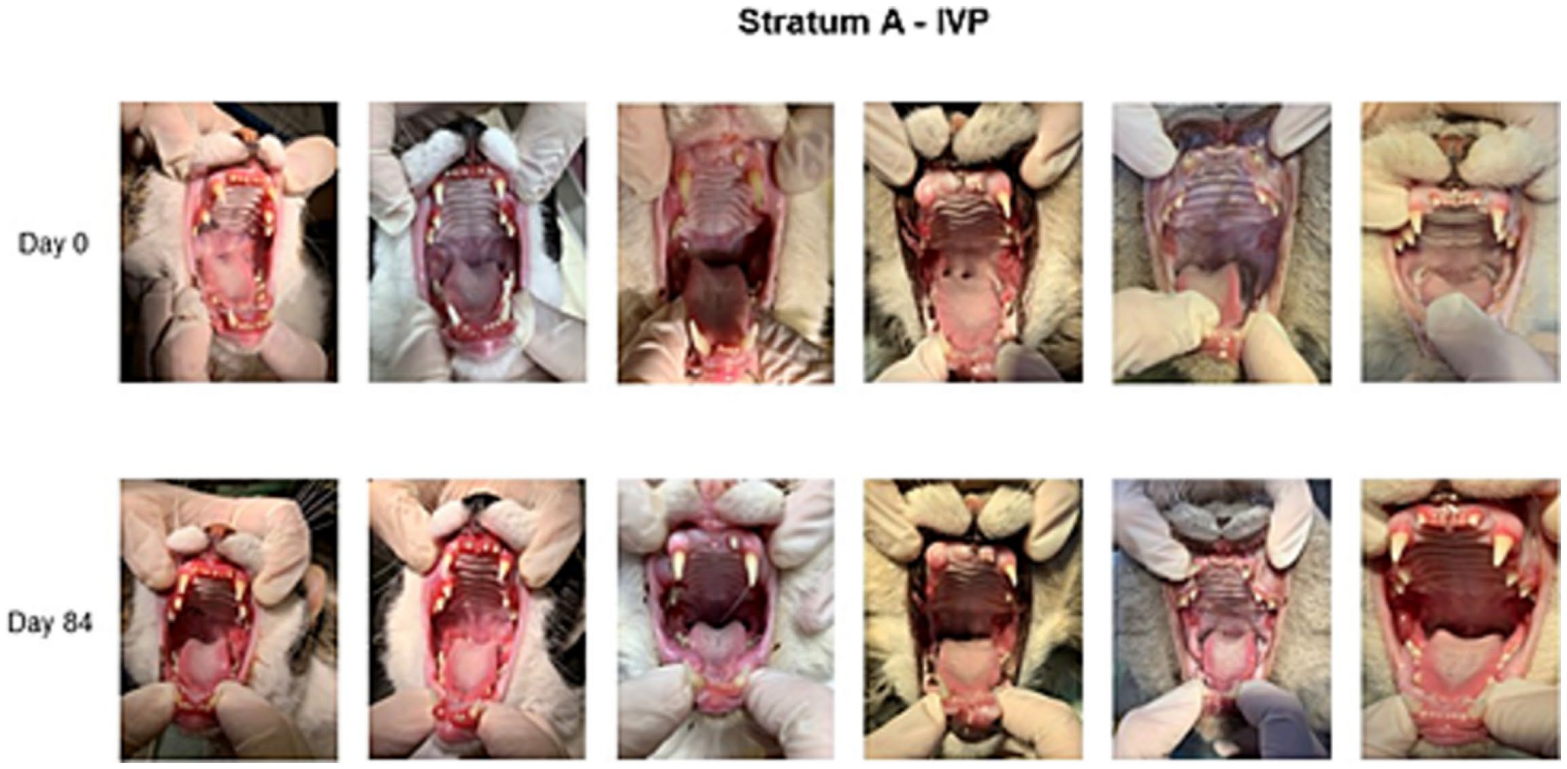
Pictures of the oral cavities taken on day 0 and day 84 in the cats treated with the IVP in stratum A.

**Figure 3:**
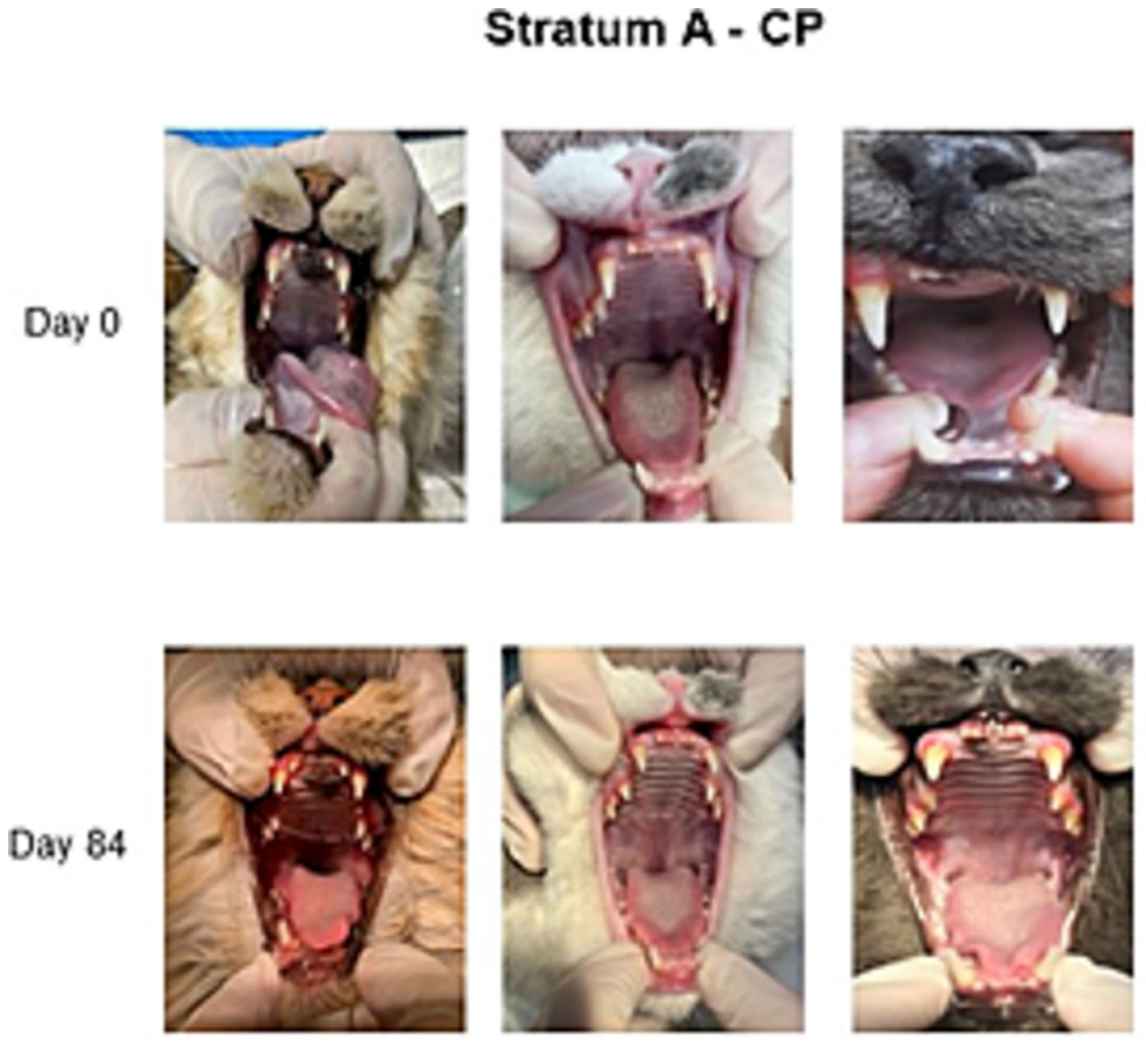
Pictures of the oral cavities taken on day 0 and day 84 in the cats treated with the CP in stratum A.

**Figure 4:**
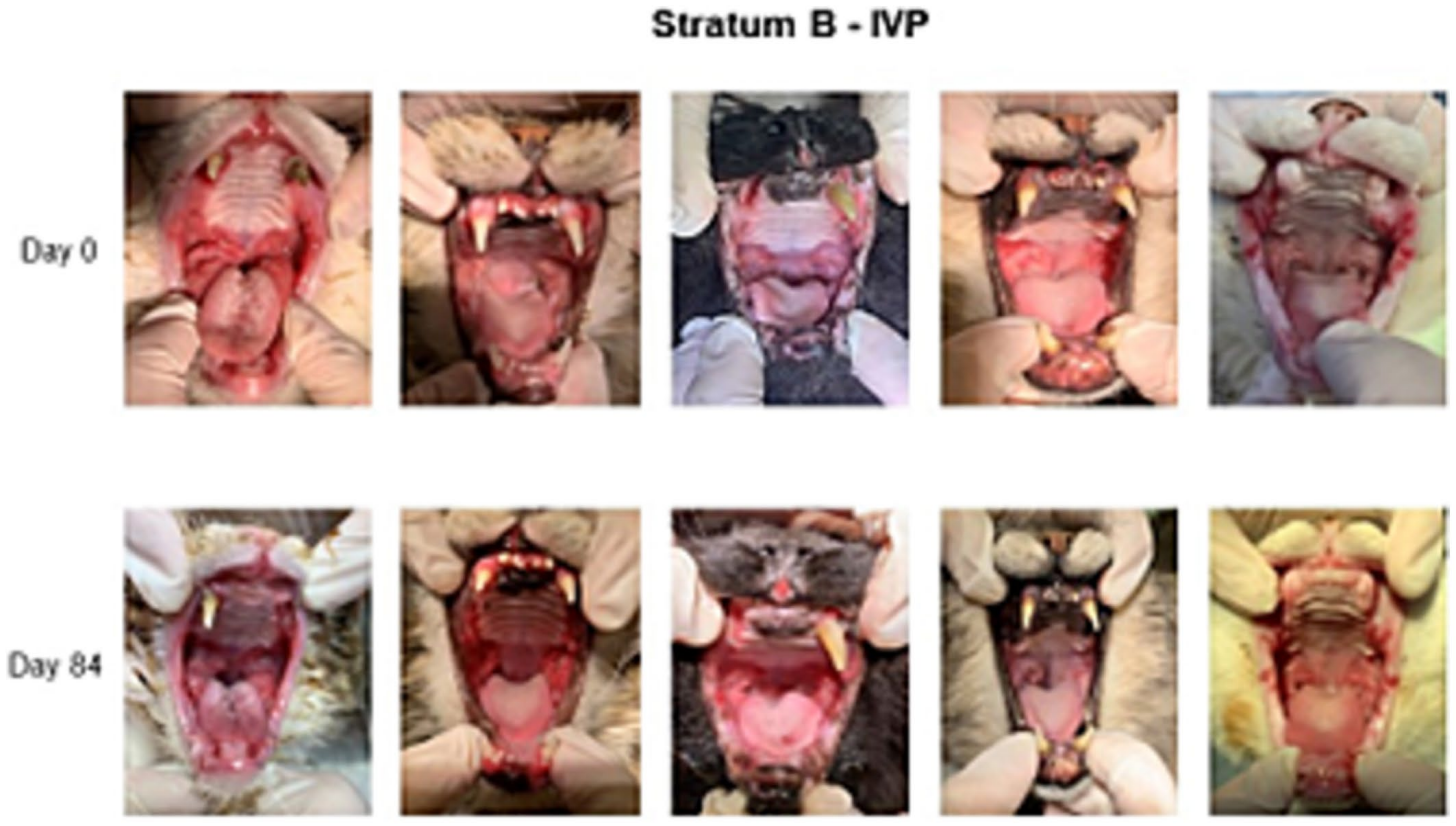
Pictures of the oral cavities taken on day 0 and day 84 in the cats treated with the IVP in stratum B.

**Figure 5:**
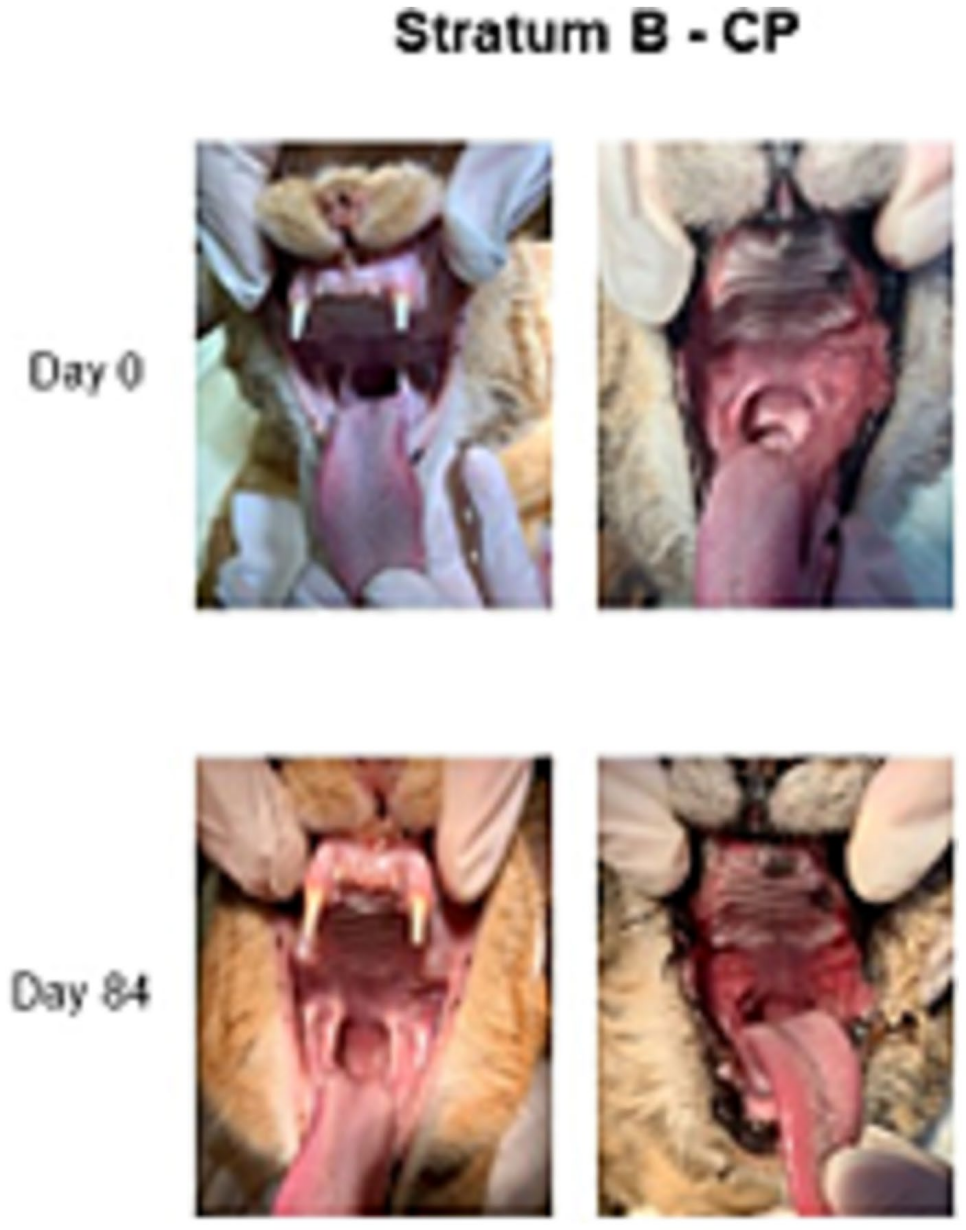
Pictures of the oral cavities taken on day 0 and day 84 in the cats treated with the CP in stratum B.

When assessing the pictures of the oral cavity at day 0 versus day 84, it was clear that minimal changes over time were observed (see Figures 2–5; Table 10).

#### Failure time

There was no significant difference in failure time between the two treatment groups (*p* = 0.629).

#### Days without rescue therapy

Three animals, all in the treated group, received concomitant medication. There was no significant difference between the two treatment groups in the percentage of days without rescue therapy (*p* = 0.26).

#### Harley’s biopsy score change

**Figure 6:**
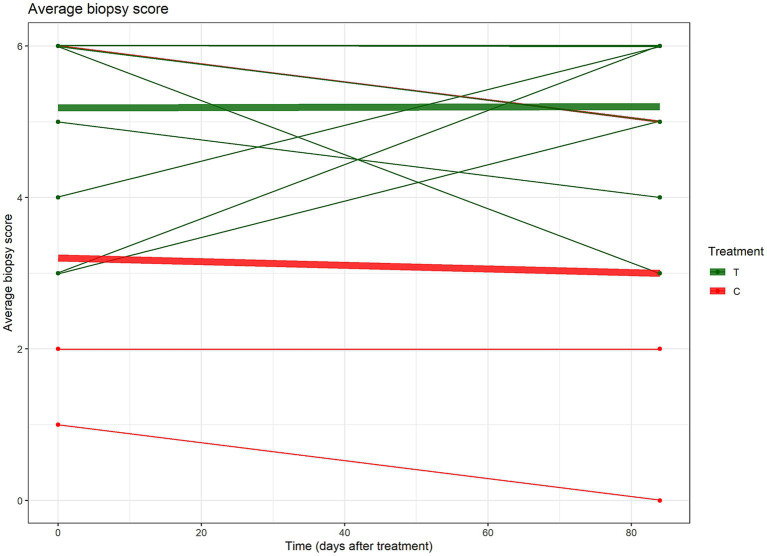
Biopsy score as a function of treatment and time. Thin lines represent individual scores, whereas thick lines are the means per treatment group.

There was no significant difference in Harley score (sum of left and right biopsy reading) change from day 0 to day 84 between the two treatments (*p* = 0.332, Figure 6 and Table 11).

**Table 11:** Biopsy score as a function of treatment and time, and change from baseline without and with LOCF.

	Time after treatment					
	0		84 ± 3		Mean	
	CP	IVP	CP	IVP	CP	IVP
Biopsy score						
Mean	3.2	5.2	3.0	5.2	2.9	5.2
Stand. error	1.2	0.4	1.2	0.3	1.1	0.2
Min	1.0	3.0	0.0	3.0	0.5	4.0
Max	6.0	6.0	5.0	6.0	5.5	6.0
Biopsy score LOCF						
Mean	3.2	5.2	3.0	5.3	3.1	5.2
Stand. error	1.2	0.4	1.0	0.3	1.0	0.2
Min	1.0	3.0	0.0	3.0	0.5	4.0
Max	6.0	6.0	5.0	6.0	5.5	6.0

	CP	IVP	CP	IVP
Biopsy score Δ Baseline				
Mean	−0.8	−0.1	/	/
Stand. error	0.3	0.6	/	/
Min	−1.0	−3.0	/	/
Max	0.0	3.0	/	/
*p*-value	0.332		/	/
Biopsy score Δ Baseline LOCF				
Mean	−0.2	−0.1	/	/
Stand. error	0.6	0.5	/	/
Min	−1.0	−3.0	/	/
Max	2.0	3.0	/	/
*p*-value	0.594		/	/

#### Owner report

**Figure 7:**
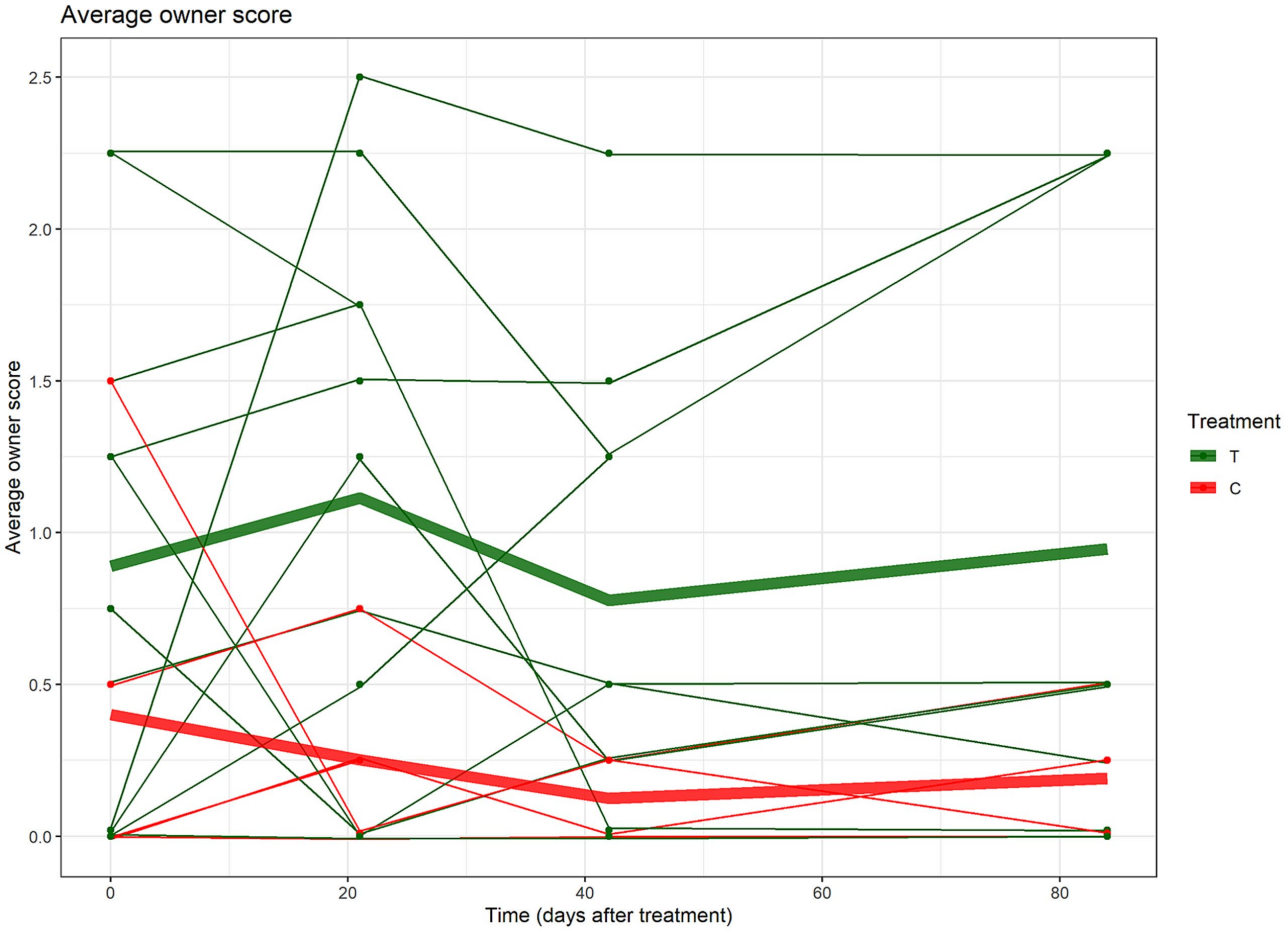
Average owner score as a function of treatment and time. Thin lines represent individual scores, whereas thick lines are the means per treatment group.

For the change of the average score from baseline with LOCF applied and using non-parametric longitudinal data analysis, no significant differences were found between the two treatment groups (*p* = 0.762, Figure 7; Table 12), for the effect of time (*p* = 0.095) or for the interaction (*p* = 0.984).

**Table 12:** Average owner score as a function of time and treatment and change from baseline without and with last observation carried forward (LOCF).

	Time after treatment									
	0		21 ± 3		42 ± 3		84 ± 3		Mean	
	CP	IVP	CP	IVP	CP	IVP	CP	IVP	CP	IVP
Owner score										
Mean	0.4	0.9	0.3	1.1	0.1	0.8	0.2	1.0	0.2	1.0
Stand. error	0.3	0.3	0.1	0.3	0.1	0.2	0.1	0.3	0.1	0.2
Min	0.0	0.0	0.0	0.0	0.0	0.0	0.0	0.0	0.0	0.0
Max	1.5	2.3	0.8	2.5	0.3	2.3	0.5	2.3	0.5	2.0
Owner LOCF										
Mean	0.4	0.9	0.3	1.1	0.2	0.9	0.2	1.1	0.3	1.0
Stand. error	0.3	0.3	0.1	0.3	0.1	0.2	0.1	0.3	0.1	0.2
Min	0.0	0.0	0.0	0.0	0.0	0.0	0.0	0.0	0.0	0.0
Max	1.5	2.3	0.8	2.5	0.3	2.3	0.5	2.3	0.5	2.0

	CP	IVP	CP	IVP	CP	IVP	CP	IVP
Owner Δ Baseline								
Mean	−0.2	0.2	−0.4	0.0	−0.3	0.1	−0.2	0.1
Stand. error	0.3	0.3	0.3	0.3	0.4	0.4	0.3	0.3
Min	−1.5	−1.2	−1.3	−1.5	−1.5	−1.5	−1.4	−0.9
Max	0.3	2.5	0.0	2.2	0.3	2.2	0.3	2.3
*p*-value	0.683		0.520		0.814		0.909	
Owner Δ Baseline LOCF								
Mean	−0.2	0.2	−0.3	0.0	−0.2	0.2	−0.2	0.1
Stand. error	0.3	0.3	0.3	0.3	0.3	0.3	0.3	0.3
Min	−1.5	−1.2	−1.3	−1.5	−1.5	−1.5	−1.4	−0.9
Max	0.3	2.5	0.3	2.2	0.3	2.2	0.3	2.3
*p*-value	0.683		0.954		0.909		0.909	

## Discussion

Eleven cats were treated intravenously with the IVP, and five cats received an intravenous injection with the CP. All study participants were controlled for adverse events. Two serious adverse events occurred: one animal in the IVP group and one animal in the CP died. The death of the animals is believed not to be related to the administration of the IVP/CP. For most general clinical sign variables considered, few events were found in both groups. For none of the considered variables, significantly more events were found in the IVP group compared to the CP group. This is in line with a safety study using xenogeneic equine PB-MSCs systemically in cats (17).

It needs to be mentioned, though, that xenogeneic application in dogs did result in the generation of xeno antibodies after repeated injections with the same dose (0.3×10^6^) used in the current study (19). In the current study, a single intravenous application was used, so further research needs to clarify whether repeated injections would result in a similar response in cats compared to the latter study in dogs. For the blood and serum chemistry variables, the distribution of observations out of limits was similar in the treatment and control groups, and the treatment and control groups did not differ significantly from each other. Moreover, the immunomodulatory properties of ePB-MSCs on feline PBMCs (17) were in line with the data reported on canine PBMCs (19). This implies similar mode of action capacities in both animal species and may modulate a potential humoral immune response after repeated injections. Further research is warranted to provide more information about potential immune recognition after repeated injections at higher doses. In addition, passage 10 ePB-MSCs were used in the current study, and it has been reported that P9 bone marrow-MSCs had significantly increased production of tumor necrosis factor alpha (TNF-α), (IL-1β), and (IL-10) compared to P3 MSCs (20). Whether this is the case for ePB-MSCs and the clinical relevance remains to be investigated.

For the SDAI, a significant overall effect was found, and also at 42 days, with CP having a significantly lower score compared to IVP. However, the average score at baseline was also substantially lower in the CP group compared to the IVP group. When considering the change from baseline, all significant effects disappeared. Along the same lines, a significantly lower biopsy score was found for CP as compared to IVP at 84 days when applying LOCF, but this effect disappeared completely when considering the change from baseline. For the average owner-reported score, a significant overall effect was found, with CP having a significantly lower score compared to IVP. However, the average score at baseline was also substantially lower in the CP group compared to the IVP group. When considering the change from baseline, all significant effects disappeared. It needs to be mentioned that the considerable differences in the baseline scores between animals and treatment groups indicate the importance of stratification into different subgroups at baseline and show its importance for future studies, including larger numbers. This is in contrast with autologous and allogeneic adipose-derived mesenchymal stem cell (ASC) studies reporting clinical benefits in FCGS (5, 6, 12, 21, 22). It needs to be mentioned, though, that it is very challenging to compare a xenogeneic low dose (0.3 ×10^6^ cells) single injection study with allogeneic or autologous treatments with a significantly higher (20 ×10^6^) cell number, where cats were treated twice, with 1 month in between.

In this study, the efficacy of the MSCs was evaluated at 3 months, and no significant effect was seen between the IVP and the CP. However, in the study reported by Arzi et al. (12) in which ASCs were administered to cats with chronic, non-responsive gingivostomatitis, the clinical response was observed between 3 and 6 months after ASCs administration. Thus, the evaluation of the efficacy of the treatment in this study might be too early as well. Moreover, the cats suffered a few days longer from the lesion incision closing than expected after the biopsy procedures, which might also have influenced the healing time. Therefore, in future studies, it might be more interesting to evaluate the efficacy of the treatment 6 months after MSCs administration.

## Conclusion

In conclusion, the single intravenous application of equine peripheral blood-derived mesenchymal stem cells in cats suffering from chronic gingivostomatitis was safe. Based on the current data using the current study design, there was no evidence that the intravenous application of equine peripheral blood-derived mesenchymal stem cells was better than control. Further studies evaluating the efficacy of equine peripheral blood-derived mesenchymal stem cells in cats suffering from chronic gingivostomatitis with a longer follow-up, different evaluation parameters, and/or adjusted treatment regimen are recommended.

## Data Availability

The raw data supporting the conclusions of this article will be made available by the authors, without undue reservation.
